# Análise do efeito da alocação de mamógrafo sobre indicadores de saúde da mulher

**DOI:** 10.1590/0102-311XPT220122

**Published:** 2024-07-29

**Authors:** Alana Ramos da Silva, Alexandre Chibebe Nicolella, Elaine Toldo Pazello

**Affiliations:** 1 Faculdade de Economia, Administração e Contabilidade de Ribeirão Preto, Universidade de São Paulo, Ribeirão Preto, Brasil.

**Keywords:** Câncer de Mama, Rastreamento, Mamografia, Saúde da Mulher, Breast Cancer, Screening, Mammography, Women’s Health, Cáncer de Mama, Tamizaje, Mamografía, Salud de la Mujer

## Abstract

A detecção precoce de câncer de mama permite formas de tratamentos mais eficazes. Entretanto, o acesso generalizado à principal ferramenta de rastreamento, a mamografia, ainda é um desafio para o sistema público de saúde brasileiro. Este estudo tem o objetivo de analisar o efeito da alocação de mamógrafos sobre indicadores de saúde da mulher. Em 2013, dentre os 4.557 municípios que não tinham o equipamento, 260 receberam até 2019. A principal hipótese é que o efeito de receber o mamógrafo seja heterogêneo entre as localidades e que receber o equipamento dependa de variáveis observáveis (pareamento por escore de propensão) e não observáveis (modelo de efeitos fixos). Os resultados indicam que os municípios brasileiros que tiveram mamógrafo em uso a partir de 2014 obtiveram aumentos na realização de exames, porém sem efeitos de curto prazo para diagnósticos e óbitos por neoplasia maligna da mama. Além de equipamentos, uma estrutura mais complexa que envolve outros fatores como acesso a consultas, profissionais qualificados, tempo de espera etc. são importantes para melhorar os indicadores de saúde femininos no recorte de municípios analisados.

## Abstract (en)

The early detection of breast cancer enables more effective forms of treatment. However, widespread access to its main screening tool, mammography, remains a challenge for the Brazilian public health system. This study aimed to analyze the effect of allocating mammography equipment on women’s health indicators. In 2013, of the 4,557 municipalities that lacked the equipment, 260 received it up to 2019. The main hypothesis of this study suggests that receiving the mammography device would show a heterogeneous effect between locations and that such receival would depend on observable (propensity score matching) and non-observable variables (fixed effects model). Results indicate that the Brazilian municipalities that had mammography equipment in use from 2014 onward increased their number of exams without short-term effects to diagnoses and deaths due to malignant breast neoplasia. In addition to equipment, a more complex structure involving other factors (such as access to consultations, qualified professionals, waiting time, etc.) is important to improve women’s health indicators in the analyzed municipalities.

## Resumen

La detección temprana del cáncer de mama permite formas de tratamientos más eficaces. Sin embargo, el acceso generalizado a la principal herramienta de seguimiento, la mamografía, sigue siendo un desafío para el sistema público de salud brasileño. Este estudio tiene como objetivo analizar el efecto de la asignación de mamógrafos en los indicadores de salud de la mujer. En 2013, de los 4.557 municipios que no tenían el equipo, 260 lo recibieron hasta 2019. La hipótesis principal es que el efecto de recibir un mamógrafo es heterogéneo entre las localidades y que recibir el equipo depende de variables observables (emparejamiento por puntaje de propensión) y no observables (modelo de efectos fijos). Los resultados indican que en los municipios brasileños que han tenido mamógrafo en uso a partir de 2014 obtuvieron aumentos en la realización de exámenes, pero sin efectos a corto plazo sobre los diagnósticos y las muertes por neoplasia maligna de la mama. Además de los equipos, una estructura más compleja que involucra otros factores como el acceso a consultas, profesionales calificados, tiempo de espera, etc. son importantes para mejorar los indicadores de salud de las mujeres en el recorte de municipios analizados.

## Introdução

O câncer de mama é o tipo de câncer mais diagnosticado em mulheres e se caracteriza como uma doença heterogênea com grande variação em suas características e manifestações clínicas ^1^. No Brasil, de 2023 a 2025 são esperados cerca de 73.610 novos casos. É a primeira causa de morte por câncer em mulheres no país e a segunda em incidência ^1^.

Apesar do alto número de casos por ano, o diagnóstico precoce do câncer de mama eleva as taxas de sobrevivência das mulheres. Em geral, as possibilidades de tratamento e sobrevivência dependem basicamente do estágio em que o tumor foi descoberto ^2^. As ações e políticas de prevenção ao câncer de mama auxiliam na redução dos custos de tratamento futuros e na melhora da qualidade de vida das mulheres ^3^.

O diagnóstico precoce pode minimizar custos emocionais, financeiros e físicos de um diagnóstico de câncer e quaisquer tratamentos subsequentes ^4^. Entre os impactos negativos do diagnóstico tardio da paciente é possível citar a depressão e a ansiedade, a perda de produtividade no trabalho, o aumento das despesas de saúde, a redução da expectativa de vida e a mortalidade precoce ^5^. No entanto, os benefícios da mamografia associados à redução da mortalidade devem ser ponderados por conta de resultados falsos positivos, sobrediagnóstico e sobretratamento ^2^ ^,^ ^4^.

As principais estratégias para a detecção do câncer de mama são: (1) o diagnóstico precoce que ocorre a partir dos sintomas iniciais da doença, tais como nódulos mamários ou alterações e lesões na pele da mama; e (2) o rastreamento, que se resume na aplicação de exames em uma população sem sinais sugestivos da doença para identificar alterações e encaminhar resultados anormais para a investigação diagnóstica ^6^.

Dessa maneira, o rastreamento aumenta as chances de detecção de um câncer em estágio inicial, ampliando a sobrevivência das mulheres. O método mais comum de diagnóstico precoce é a mamografia, um exame realizado por meio de mamógrafo que realiza imagens de raios-X de baixa intensidade das mamas para identificar anomalias ^2^. As mamografias de rastreamento são indicadas às mulheres assintomáticas na faixa etária de 50 a 69 anos de idade com periodicidade de dois anos, enquanto a mamografia diagnóstica é indicada para avaliação de lesões mamárias suspeitas em qualquer idade ^6^. A justificativa para a ampliação do acesso generalizado à mamografia é que a detecção precoce de cânceres potencialmente fatais permite formas de tratamentos mais eficazes ^4^.

Estudos clínicos randomizados e de caso-controle que acompanham mulheres ao longo do tempo concluíram que as mamografias de rastreamento implicam em uma redução significativa da mortalidade por câncer de mama ao detectar tumores em estágios iniciais ^7^ ^,^ ^8^ ^,^ ^9^ ^,^ ^10^. Tais estudos geraram recomendações de sociedades médicas, órgãos de saúde e políticas públicas acerca da relevância do aumento das mamografias realizadas ^2^. No entanto, o acesso generalizado a esse exame não é uma realidade no contexto da saúde pública brasileira. A cobertura de mamografias é mais alta nas microrregiões com menor desigualdade de renda e com maior acesso a programas de saúde pública ^11^. Além disso, a cobertura está diretamente relacionada à disponibilidade de mamógrafos entre as microrregiões ^12^.

A desigualdade no acesso à realização desse exame no Brasil ainda é um grande desafio ^13^. No âmbito do Sistema Único de Saúde (SUS) é possível identificar uma baixa cobertura para as mulheres que compõem o grupo alvo. Em 2012, apenas 43,7% dos exames esperados foram efetivamente realizados ^12^.

A cobertura do exame é maior nos grandes centros urbanos, que também são as localidades com menor desigualdade social ^11^. Entre os principais fatores que afetam a realização de mamografia pelas mulheres estão melhores condições socioeconômicas e residência em regiões mais desenvolvidas ^5^. A probabilidade de realizar o exame se amplia com a renda familiar e a escolaridade e é reduzida conforme aumenta a distância entre a moradia da paciente e a unidade de radiologia mais próxima ^14^.

Além disso, a cobertura e a disponibilidade de mamógrafos é bastante heterogênea. Em 2012, 49,3% das Regiões de Saúde apresentavam baixa cobertura de exames e baixo grau de utilização de equipamentos ^12^. Entre 2011 e 2012, as regiões Norte e Nordeste apresentavam o menor número de mamógrafos disponíveis ^3^, bem como menor quantidade de radiologistas ^11^. Da mesma forma, em 2013, as mulheres residentes nas regiões Sudeste e Sul, brancas e com planos de saúde privados tinham maior acesso ao rastreamento do câncer de mama ^15^.

Além do entrave decorrido da desigualdade de acesso aos exames, a qualidade dos equipamentos também é um fator relevante. Estudos mostram uma associação entre radiação emitida pelos aparelhos de radiologia e o maior risco para câncer de mama. Dessa maneira, o Programa Nacional de Qualidade em Mamografia (PNQM) tem o intuito de garantir a qualidade dos exames de forma a minimizar os riscos associados aos raios-X ^16^. Sendo assim, as desigualdades sociais e geográficas na acessibilidade e na qualidade dos mamógrafos comprometem a detecção precoce do câncer de mama ^14^.

Ademais, outros condicionantes são cruciais para a expansão do acesso e da qualidade das mamografias. Em especial, os recursos humanos envolvidos no processo de solicitação e interpretação dos exames de imagens, além da operacionalização e manutenção dos equipamentos ^12^. Por outro lado, a disseminação de informações a respeito da importância da mamografia para o público-alvo se mostra importante ^12^, além do aumento da oferta de exames e maior interiorização dos equipamentos, políticas de inclusão social e de aumento da renda também são essenciais ^14^.

Com relação à disponibilidade de mamógrafos em uso, há uma lacuna na literatura a respeito do processo de interiorização desses equipamentos. Uma possibilidade é que uma política de alocação em locais distintos pode ter efeitos totalmente diversos entre as localidades contempladas, o que amplia a importância de entender em quais municípios o mamógrafo atende o maior número de mulheres, visando a uma política de saúde mais efetiva.

Dessa forma, o objetivo deste artigo é estimar o efeito da alocação de mamógrafos sobre a realização de mamografias de rastreamento, diagnósticos e mortalidade por câncer de mama em mulheres de 50 a 69 anos em municípios que não tinham mamógrafo em uso no ano de 2013 e passaram a ter o equipamento entre os anos de 2014 a 2019. A principal hipótese é que o efeito da alocação do equipamento é heterogêneo entre as localidades. O que implica que municípios com menor infraestrutura de saúde podem utilizar de forma menos eficiente o equipamento médico, comparativamente aos municípios com um sistema de saúde organizado. Ao observar os municípios a partir de 2013, este estudo analisa uma política de expansão de equipamentos em locais específicos com pouca infraestrutura de saúde, considerando que foram beneficiados pelo mamógrafo mais recentemente.

O tema tem sido relevante na literatura devido à importância da criação de políticas públicas que visam à redução da incidência e das taxas de mortalidade por câncer de mama. A maior parte dos estudos encontram o efeito médio ao analisar a cobertura do rastreamento da doença ^11^ ^,^ ^12^ e a associação entre o acesso aos exames de mamografia e características geográficas e populacionais buscando examinar as desigualdades de acesso aos exames ^3^ ^,^ ^14^ ^,^ ^15^. Este estudo contribui com a literatura existente ao analisar o efeito causal da alocação de mamógrafos em municípios específicos que receberam o equipamento sobre indicadores da saúde das mulheres que compõem o público-alvo e inova ao fazer uso de recursos econométricos para avaliar o impacto da alocação do equipamento em localidades que não o tiveram até então. Estudos como este são importantes para analisar a efetividade da alocação de equipamentos em localidades com menor porte populacional e com pouco acesso a serviços de saúde de média e alta complexidades, isto é, os que mais necessitam de políticas públicas de acesso generalizado a exames e tratamentos de saúde.

## Métodos

### Estratégia empírica

A literatura destaca a importância da expansão territorial de equipamentos visando à melhora da cobertura e do acesso aos exames de mamografia pela população feminina ^3^ ^,^ ^11^ ^,^ ^12^ ^,^ ^15^. Dessa maneira, busca-se analisar se a atribuição de mamógrafos no SUS em municípios específicos que não tinham o equipamento em 2013 causa impacto sobre os indicadores de saúde da mulher. O que permite descobrir se o efeito causal é similar aos que a literatura encontra para a média de municípios em relação à cobertura de mamografias ^11^ ^,^ ^12^ ^,^ ^14^.

O principal problema para essa especificação é definido pela literatura econométrica por endogeneidade. Em decorrência do fato de que a escolha dos municípios que são contemplados com equipamento não ocorre de forma aleatória pelos gestores públicos, existem diferenças em características observáveis e não observáveis entre os municípios que receberam ou não o mamógrafo. Em relação às variáveis observáveis, os municípios que já têm equipes e locais de saúde especializados podem ter mais facilidade em recebê-los e disponibilizá-los ao público-alvo do que localidades que não dispõem dessas características. Além disso, o efeito causal do mamógrafo pode ser atribuído não pelo equipamento em si, mas pelo melhor sistema de saúde de uma determinada localidade, por exemplo, mais médicos e hospitais.

A literatura justifica o uso da metodologia de pareamento por escore de propensão, desde que a hipótese de seleção por observáveis possa ser assegurada, para permitir uma comparação adequada entre os municípios que receberam (grupo de tratamento) e não receberam o mamógrafo (grupo de controle) por meio de covariáveis que predizem o recebimento dessa política de alocação. O método de pareamento utilizado é o vizinho mais próximo com reposição 1:1 em que cada unidade tratada é pareada com pelo menos uma unidade de controle com as características mais próximas possíveis ^17^. Foi utilizado a calibragem de 0,01, ou seja, essa foi a maior distância tolerada entre as probabilidades estimadas para tratamento e controle de receber o equipamento. Devido ao número limitado de municípios tratados, a reposição permite que cada unidade de controle possa ser reutilizada e combinada com mais de uma unidade tratada. Dessa maneira, o pareamento torna mais plausível a hipótese de tendências paralelas, ou seja, a suposição de que os municípios descendem de trajetórias parecidas.

Tendo em vista as variáveis disponíveis e a tomada de decisão dos gestores públicos acerca da alocação de equipamentos em municípios mais prioritários, foram consideradas as seguintes covariáveis de pré-tratamento em 2010: renda *per capita*, números de médicos ginecologistas, de clínicos gerais, de técnicos e auxiliares de radiologia, de hospitais gerais e de mulheres de 50 a 69 anos. Acredita-se que essas covariáveis são determinantes para a alocação de mamógrafos, devido à necessidade de uma infraestrutura de recursos físicos e humanos para o pedido do exame e instalação, manutenção e uso do equipamento ^12^ ^,^ ^14^. Por exemplo, municípios com maior estrutura de saúde podem precisar de mais equipamentos para a realização de exames e terem maiores chances de receber o mamógrafo.

A segunda fonte de endogeneidade é a omissão de variáveis não observadas que podem enviesar as estimativas do efeito médio da participação em um programa. A literatura descreve algumas especificações para lidar com esse problema ^18^ ^,^ ^19^ ^,^ ^20^. Ao assumir que o ano em que a localidade é contemplada pelo equipamento não faz diferença no efeito dessa política e que esse efeito é igual para os municípios selecionados em 2013 sobre os indicadores de saúde, é possível utilizar uma das técnicas mais comuns utilizada em economia para medir o efeito de um tratamento: a regressão linear com efeitos fixos de tempo e grupo ^21^.

Além de lidar com o problema da endogeneidade causado por variáveis não observáveis, o painel consegue estimar o efeito homogêneo do equipamento no recorte de municípios analisados. Sendo assim, essa especificação tenta controlar características não observáveis dos municípios que são fixas ao longo do tempo e lida com a natureza dos dados disponíveis, caracterizada por um número pequeno de localidades que receberam o mamógrafo a cada ano a partir de 2014 e a alta quantidade de observações zeradas para diagnósticos e óbitos.

Dessa forma, a análise realizada aqui consiste em estimar o efeito causal da alocação de mamógrafos, ao longo do recorte temporal de 2013 a 2019, nos municípios que não dispuseram do equipamento em 2013. Esse período é analisado, pois as variáveis de interesse estão disponíveis juntas a partir do ano de 2013 no Departamento de Informática do SUS (DATASUS) e 2020 foi excluído devido ao impacto da pandemia sobre assistência à saúde.

Os indicadores de saúde da mulher aqui analisados são: (1) exames de mamografia de rastreamento; (2) diagnósticos de neoplasia maligna da mama; e (3) óbitos por neoplasia maligna da mama. Tais indicadores são considerados importantes para analisar a cobertura dos exames realizados no país, bem como para alocação de recursos para o tratamento e diagnóstico precoce do câncer mais comum entre a população feminina.

Seguindo a recomendação do Instituto Nacional de Câncer (INCA), a análise se direciona ao rastreamento do câncer de mama, isto é, às mulheres de 50 a 69 anos assintomáticas. A partir de indicadores municipais de saúde que variam ao longo do período de 2013 a 2019, é possível definir a seguinte equação de regressão:

$${Saúde}_{it}=\alpha +\beta_{1}{equip}_{it}+{Município}_{i}+{Ano}_{t}+{Χ}_{it}+u_{it}$$( 1 )

em que *Saúde*_*it*_ representa um indicador de saúde (exames realizados, diagnósticos ou óbitos por neoplasia maligna da mama) por 100 mil mulheres de 50 a 69 anos do município de residência *i* durante o ano *t*; *equip*_*it*_ é uma variável categórica que recebe 1 caso o município *i* tenha mamógrafo em uso durante o ano *t*; *Munícipio*_*i*_ são *dummies* de efeitos fixos de municípios; *Ano*_*t*_ são *dummies* para os efeitos fixos de tempo; *X*_*it*_ é um vetor de variáveis de saúde pública (números de médicos ginecologistas, de médicos clínicos gerais e de técnicos e auxiliares de radiologia que atendem no SUS) de 2013 a 2019; e *u*_*it*_ é o termo de erro.

### Fonte de dados

Os dados de saúde foram obtidos pelos sistemas de informação disponíveis no DATASUS. A plataforma também permite a exploração de informações demográficas e socioeconômicas, particularmente dados censitários e estimativas do Instituto Brasileiro de Geografia e Estatística (IBGE).

Dessa forma, foram obtidos os indicadores em nível municipal por residência do período de 2013 a 2019. Das variáveis de resultado, as quantidades dos exames de mamografia de rastreamento foram obtidas no Sistema de Informações Ambulatoriais do SUS (SIA-SUS), os diagnósticos e óbitos por neoplasia maligna da mama pelo Painel Oncologia e Sistema de Informações sobre Mortalidade (SIM), respectivamente.

A variável de tratamento, mamógrafos em uso, foi calculada a partir da média anual verificada no Cadastro Nacional de Estabelecimentos de Saúde (CNES-Recursos Físicos). A renda *per capita* em 2010 e as projeções para a população de mulheres de 50 a 69 anos entre 2013 e 2019 foram obtidos pelos indicadores demográficos e socioeconômicos do IBGE. Para as informações sobre os números de médicos ginecologistas, de médicos clínicos gerais e de técnicos e auxiliares de radiologia, foram calculadas as médias anuais do CNES-Recursos Humanos e a quantidade média de hospitais gerais verificada no CNES-Recursos Físicos.

## Resultados

### Estatísticas descritivas

Dos 5.565 municípios brasileiros com informações disponíveis, 4.557 não dispunham de equipamento para a realização de mamografias em 2013. Desses, 260 foram equipados com pelo menos um mamógrafo a partir de 2014. A Tabela 1 destaca um comparativo socioeconômico entre os municípios brasileiros e os que compõem a amostra utilizada nas estimativas. Os indicadores apresentados na tabela se referem ao ano de 2010, pré-tratamento, e mostram as diferenças nas características observáveis entre os municípios.

**Tabela 1:** Comparativo entre os municípios brasileiros, municípios com mamógrafo, amostra selecionada e unidades tratadas.

Características	Brasil	Municípios com mamógrafo (2013)	Amostra selecionada (sem mamógrafo em 2013)	Municípios tratados (com mamógrafo a partir de 2014)
População média (habitantes)	34.570	135.084	12.336	27.859
Densidade demográfica média (habitantes/km^2^)	109,1	435,9	36,8	92,2
Renda *per capita* (R$)	12.602,48	18.341,46	11.333,03	13.318,39
Proporção de municípios com grande ou média concentração urbana (%)	11,8	36,8	6,3	14,6
Proporção de municípios nas regiões Nordeste e Norte (%)	40,3	29,5	42,7	59,2
Proporção de municípios nas regiões Sul, Sudeste e Centro-oeste (%)	59,7	70,5	57,3	40,8
Municípios	5.565	1.008	4.557	260

Fonte: elaboração própria a partir das estimativas populacionais e produto interno bruto dos municípios pelo Instituto Brasileiro de Geografia e Estatística ^30^.

Nota: dos 5.570 municípios brasileiros, cinco apresentaram dados faltantes em 2010 e foram retirados da análise.

A primeira coluna apresenta os valores médios das características para todos os municípios brasileiros; a segunda, para as localidades que dispunham de mamógrafo em uso em 2013; a terceira, para as localidades que não dispunham de mamógrafo em uso em 2013; e, por fim, a quarta para as unidades tratadas, ou seja, para aquelas que foram contempladas com pelo menos um equipamento a partir de 2014.

É possível verificar que os municípios selecionados - dados da terceira coluna - têm população, densidade demográfica e renda *per capita* menores que o total de municípios brasileiros. Outra característica das unidades da amostra selecionada é o fato de não abranger capitais brasileiras e ter uma proporção pequena de municípios com grande ou média concentração urbana, em comparação a proporção nacional. O grupo de municípios que já dispunham de equipamentos em 2013 se caracteriza por localidades com maior concentração populacional urbana residente no centro-sul do país e maior renda *per capita*. Ademais, ao se analisar as unidades tratadas, observa-se que as regiões Norte e Nordeste foram as que proporcionalmente receberam mais equipamentos a partir de 2014.

É importante ressaltar que as variáveis da Tabela 1 não fazem parte do modelo estimado, apenas foram usadas para caracterizar e obter um perfil geral dos grupos de municípios analisados durante o período de 2013 a 2019. Como é possível perceber da análise da Tabela 1, a alocação de mamógrafos não ocorre de forma aleatória, justificando o uso da técnica de pareamento.

Entre a amostra de municípios selecionados, a Tabela 2 apresenta estatísticas descritivas dos grupos de tratamento (municípios que receberam o equipamento entre 2014 e 2019) e de controle (não receberam equipamento no período) das variáveis de interesse por 100 mil mulheres de 50 a 69 anos: exames, diagnósticos e óbitos por neoplasia maligna da mama. As colunas consideram os dados por residência durante o período de 2013 a 2019.

**Tabela 2:** Estatísticas descritivas das variáveis de resultado (tratamento e controle).

	Unidades de tratamento							Unidades de controle						
	2013	2014	2015	2016	2017	2018	2019	2013	2014	2015	2016	2017	2018	2019
Exames por 100 mil mulheres de 50 a 69 anos														
Q_1/4_	1.498	2.115	2.547	1.846	1.968	1.149	1.279	3.509	3.846	3.892	4.317	4.312	3.293	3.032
Q_2/4_	6.524	9.568	8.534	9.689	10.499	5.822	5.733	12.000	12.602	12.582	12.800	13.487	10.101	9.898
M_e_	9.271	12.937	11.356	11.874	11.806	8.497	8.536	15.167	16.225	16.061	16.397	16.552	14.043	12.554
Q_3/4_	13.002	18.818	16.292	17.239	16.929	12.253	137.756	21.868	23.594	22.744	23.110	23.593	20.030	18.557
Diagnósticos por 100 mil mulheres de 50 a 69 anos														
Q_1/4_	0,0	0,0	0,0	0,0	0,0	0,0	0,0	0,0	0,0	0,0	0,0	0,0	0,0	0,0
Q_2/4_	58,9	55,3	51,4	50,7	56,5	65,7	73,2	0,0	0,0	0,0	0,0	0,0	42,3	59,9
M_e_	84,0	76,0	69,6	69,4	68,4	81,8	87,0	87,1	83,3	80,6	84,5	81,7	96,8	108,2
Q_3/4_	124,3	119,4	111,9	110,9	113,6	129,9	123,7	138,7	134,1	125,8	134,8	131,9	154,1	174,8
Óbitos por 100 mil mulheres de 50 a 69 anos														
Q_1/4_	0,0	0,0	0,0	0,0	0,0	0,0	0,0	0,0	0,0	0,0	0,0	0,0	0,0	0,0
Q_2/4_	0,0	0,0	0,0	0,0	0,0	0,0	0,0	0,0	0,0	0,0	0,0	0,0	0,0	0,0
M_e_	20,0	35,1	30,4	24,9	26,7	24,6	30,4	24,7	22,0	25,4	26,3	26,5	26,5	27,35
Q_3/4_	31,9	47,6	46,2	35,8	46,3	39,5	47,3	0,0	0,0	0,0	0,0	0,0	0,0	0,0

Fonte: elaboração própria a partir de dados por residência do Sistema de Informações Ambulatoriais do SUS (SIA-SUS), do Painel Oncologia e do Sistema de Informações sobre Mortalidade (SIM) (2013-2019).

Nota-se, pela Tabela 2, que os quartis das unidades de tratamento dos exames de mamografia por 100 mil mulheres de 50 a 69 anos são maiores do que as medidas das unidades de controle durante todo o período de análise. Em contrapartida, os quartis dos diagnósticos e óbitos apresentam uma maior quantidade de zeros. Além disso, as médias das unidades tratadas e de controle estão mais próximas.

### Validação do pareamento

Conforme observado pelo comparativo da Tabela 1, há uma seleção não aleatória dos municípios que receberam o mamógrafo comparativamente à amostra selecionada em 2013. Por exemplo, ao verificar os municípios tratados com mamógrafo, esse grupo tem maior densidade demográfica e renda per capita. Dessa forma, o pareamento tenta tornar os municípios tratados e de controle mais parecidos entre si nas características observáveis.

A partir das etapas descritas para o pareamento por escore propensão ^17^ ^,^ ^22^ foi estimado um modelo de regressão *logit*. A variável dependente é uma *dummy* que recebe 1 caso o município recebeu o mamógrafo e 0 caso não recebeu. O modelo *logit* para os municípios apresentou os sinais esperados, tudo o mais constante: quanto menor a renda da cidade maior a probabilidade de ter o equipamento em uso. Por sua vez, quanto maior a quantidade de mil mulheres de 50 a 69 anos, médicos clínicos, técnicos e auxiliares e hospitais, maior a probabilidade do município receber o equipamento, *ceteris paribus*.

**Figura 1:**
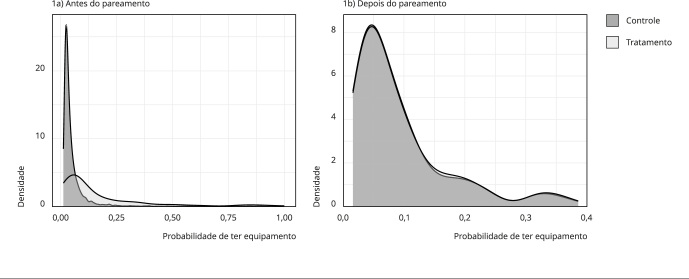
Escores de propensão antes e depois do pareamento.

O pareamento foi realizado pelo pacote *MatchIt* ^23^ do software RStudio (https://rstudio.com/) com o método do vizinho mais próximo com reposição 1:1 e calibragem de 0,01. Para verificar a qualidade do pareamento, é útil observar as distribuições de probabilidade, quanto mais similares forem as distribuições melhor terá sido o pareamento obtido. Sendo assim, a Figura 1 indica as distribuições de probabilidade para os municípios antes e depois do pareamento.

A Figura 1 destaca em cinza escuro as unidades de controle, enquanto a cor cinza clara descreve as unidades tratadas. No primeiro gráfico é possível notar que as distribuições antes do pareamento não eram semelhantes. No entanto, no segundo gráfico há semelhança entre as distribuições que ficaram visualmente sobrepostas, o que indica a similaridade entre os grupos de tratamento e controle pós-pareamento.

**Figura 2:**
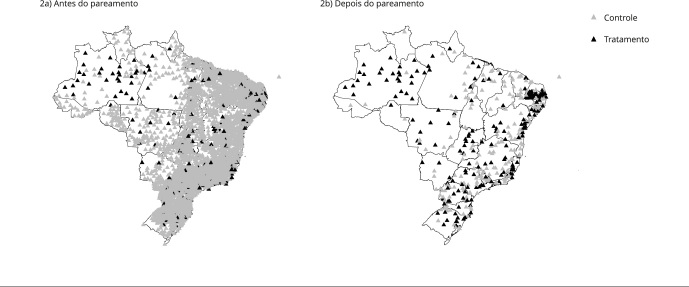
Municípios antes e depois do pareamento.

A Figura 2 destaca os municípios selecionados antes e depois do pareamento. O mapa da Figura 2a mostra todos os municípios que não tinham equipamento em 2013, identificando em preto aqueles que passaram a receber nos anos seguintes. O mapa da Figura 2b apresenta a amostra pareada, com as unidades tratadas em preto. Observa-se que, após o pareamento, há maior equilíbrio regional entre os municípios tratados e de controle.

Outra forma de verificar a qualidade do pareamento realizado ocorre por meio de testes de diferenças de médias nesses dois momentos. Os testes consideram os pesos dos escores de propensão e estão descritos na Tabela 3 com seus níveis de significância representados por asteriscos. A hipótese nula é que a diferença de média é zero entre os grupos de tratamento e controle.

**Tabela 3:** Testes de diferenças de médias das covariáveis.

Covariável (2010)	Média			Nível de significância dos testes de média
	Amostra	Tratamento	Controle	
Logaritmo natural (renda)	Antes	5,97	5,98	*
	Depois	5,92	5,93	*
Número de médicos ginecologistas	Antes	0,59	0,11	**
	Depois	0,23	0,21	*
Número de médicos clínicos gerais	Antes	7,00	2,41	**
	Depois	4,89	4,71	*
Número de técnicos/auxiliares de radiologia	Antes	1,68	0,54	**
	Depois	1,13	1,22	*
Número de hospitais gerais	Antes	0,92	0,49	**
	Depois	0,79	0,85	*
Número de mulheres de 50 a 69 anos	Antes	1.912,80	856,60	**
	Depois	1.450,62	1.422,25	*

Fonte: elaboração própria com dados do Cadastro Nacional de Estabelecimentos de Saúde (CNES) e do Instituto Brasileiro de Geogafia e Estatística (IBGE) (2010).

* Não rejeita hipótese nula;

** p < 0,01

Os testes de média indicam que não é possível rejeitar a hipótese nula nas amostras obtidas após o pareamento, e, consequentemente, todas as diferenças são estatisticamente iguais a zero após o pareamento. Tais resultados validam o pareamento por vizinho mais próximo com reposição.

### Estimações

As estimativas realizadas antes do pareamento envolveram 4.557 municípios que não tinham mamógrafo em uso em 2013, incluindo os 260 que passaram a ter durante os anos posteriores. Ao considerar os sete anos de análise (2013 a 2019), totalizou um painel de dados com 31.899 observações. Já estimativas pós-pareamento consideram uma amostra total de 431 municípios (219 unidades tratadas e 171 controles), com um painel de 3.017 observações.

A Tabela 4 apresenta os modelos de efeitos fixos de município e ano descrito na Equação 1 com seus respectivos erros-padrão em parênteses e asteriscos indicando o nível de significância. Os modelos são estimados com dados antes e após o pareamento, tendo como variáveis dependentes das estimações três indicadores de saúde por 100 mil mulheres de 50 a 69 anos: exames, diagnósticos e óbitos por neoplasia maligna da mama.

**Tabela 4:** Estimações por efeitos fixos.

	Variável dependente por 100 mil mulheres de 50 a 69 anos					
	Exames		Diagnósticos		Óbitos	
	Antes	Depois	Antes	Depois	Antes	Depois
Equipamento	1.478,03 * (624,69)	1.854,89 ** (683,46)	-3,63 (6,95)	0,27 (5,59)	2,17 (3,58)	0,69 (3,37)
Efeitos fixos de município e tempo	Sim	Sim	Sim	Sim	Sim	Sim
Controles de saúde pública	Sim	Sim	Sim	Sim	Sim	Sim
Observações	31.899	3.017	31.899	3.017	31.899	3.017
R^2^	0,017	0,030	0,006	0,008	0,000	0,007
R^2^ ajustado	-0,147	-0,135	-0,160	-0,161	-0,166	-0,162

Fonte: elaboração própria a partir de dados do Sistema de Informações Ambulatoriais do SUS (SIA-SUS), do Painel Oncologia, do Sistema de Informações sobre Mortalidade (SIM) e do Cadastro Nacional de Estabelecimentos de Saúde (CNES) (2013-2019).

Nota: estimações com dados por residência antes e depois do pareamento. Efeitos fixos de tempo e município. Controles de saúde pública: número de médicos ginecologistas, clínicos gerais e técnicos e auxiliares de radiologia do Sistema Único de Saúde (SUS).

* p < 0,05;

** p < 0,01.

Em relação às estimativas para a quantidade de exames efetuados, todos os coeficientes são positivos e significativos. A depender da coluna analisada, tudo o mais constante, a presença do equipamento ampliou entre 1.500 e 1.850 o número de exames por 100 mil mulheres de 50 a 69 anos.

Ao considerar as estimativas para os diagnósticos e óbitos por 100 mil mulheres do público-alvo, a não significância estatística em todos os modelos indica que os mamógrafos alocados nos municípios selecionados não tiveram efeito de curto prazo sobre esses indicadores de saúde.

## Discussão

Desde 2007 diversos programas e portarias têm sido implementados visando à ampliação do número de mamografias, sobretudo às mulheres de 50 a 69 anos ^24^. O Ministério da Saúde recomenda que a alocação de mamógrafos considere o acesso das mulheres, isto é, um deslocamento de 60 minutos ou 60km ^25^. Além disso, um equipamento eficiente deve realizar 6.758 mamografias/ano ^25^.

No SUS, o rastreamento do câncer de mama é predominantemente oportunístico ^26^. Dessa forma, é necessário que um profissional de saúde identifique a necessidade da mamografia e solicite o exame. O que implica que a periodicidade e o início do rastreamento sejam dependentes de decisões individuais das pacientes e dos profissionais de saúde ^26^.

Considerando o recorte de municípios analisados, os resultados destacam que o aumento de equipamentos permitiu a elevação de mamografias realizadas. O que ressalta a importância da alocação de mamógrafos nos municípios selecionados para o aumento da cobertura de mamografias entre as mulheres do público-alvo.

Conforme descrito na literatura, a cobertura de mamografias está diretamente relacionada à disponibilidade de mamógrafos em uso ^12^. As estimativas deste estudo reforçam esse resultado sobre as localidades que receberam o equipamento nos últimos dez anos.

Em relação às estimativas para os diagnósticos, a literatura médica sobre o diagnóstico de câncer descreve que a confirmação do diagnóstico de qualquer tipo de neoplasia identificada em exames clínicos, de imagens ou em ressonâncias magnéticas é realizada somente a partir de biópsia ^27^. Essa técnica consiste na análise patológica de fragmentos de nódulos ou lesões retiradas dos pacientes ^27^. Nesse sentido, acredita-se que a inclusão de equipamentos pode aumentar a detecção de alterações, porém com relação não tão direta com o diagnóstico definitivo de neoplasias malignas. Ademais, o ciclo para detecção precoce do câncer de mama necessita, além de equipamentos, de outros fatores importantes, tais como acesso a consultas, unidades básicas de saúde, equipes de saúde qualificadas, menor tempo para realização do diagnóstico definitivo ^12^, entre outros aspectos.

Da mesma forma que para diagnósticos, o resultado não significativo para os óbitos deve ser visto com cautela, tendo em vista que apenas a presença do equipamento não foi suficiente para evitar a mortalidade por câncer. Estudos descrevem a relação entre as mamografias de rastreamento e a redução da mortalidade por câncer de mama. Nesses trabalhos, são utilizadas bases de dados com observações de mulheres ao longo do tempo que realizaram e não realizaram os exames de rastreamento. No caso deste artigo, as observações são de municípios selecionados que não dispunham de mamógrafo em 2013 e passaram a ter em algum momento entre 2014 e 2019. Dessa maneira, a presença do mamógrafo em um município talvez não seja suficiente e não abrange todo o processo de rastreamento, diagnóstico definitivo e tratamento.

Portanto, as estimativas obtidas mostraram que a inclusão de equipamentos de mamografia a partir de 2014 nos municípios selecionados com pouca infraestrutura de saúde ampliaram a realização de exames, porém sem impacto de curto prazo sobre diagnósticos e óbitos por neoplasia maligna da mama. Esse trabalho contribui para a literatura ao mostrar que a alocação de mamógrafos entre municípios brasileiros pode ter efeitos distintos. Seguindo a literatura sobre o tema, é preciso não apenas ampliar os equipamentos de mamografias, mas também elevar a disseminação de informações a respeito da relevância da realização de mamografias de rastreamento ^12^. Também é necessário atenção sobre os recursos humanos e operacionais envolvidos, desde a consulta médica até a interpretação dos resultados de forma a agilizar o processo assistencial à saúde das mulheres ^12^.

Além disso, a amostra de municípios utilizada nas estimativas possui características que abrangem municípios interioranos. Estudos apontam que a realização de mamografias é maior nos grandes centros urbanos ^11^ e em localidades mais desenvolvidas ^5^, o que evidencia a necessidade de expansão de equipamentos. Em especial, a maior parte dos municípios analisados neste estudo estão localizados nas regiões Norte e Nordeste e apresentam menor densidade demográfica e poucas áreas com média e grande concentração urbana.

A literatura indica uma relação inversa entre cobertura de mamografias e condições socioeconômicas nas regiões brasileiras ^5^ ^,^ ^12^ ^,^ ^14^. Particularmente, as regiões Norte e Nordeste apresentam inadequação entre oferta e demanda da distribuição espacial de mamógrafos no SUS ^28^. Entretanto, entre 2010 e 2019, a cobertura de mamografias nas regiões Norte e Nordeste apresentou tendência de aumento até 2015 e 2017, respectivamente ^29^, sendo esse período o foco do nosso estudo.

Assim, os municípios analisados têm necessidades mais complexas para o desenho de uma política de saúde feminina específica. Dessa maneira, a expansão por si só consegue ampliar o número de mamografias, porém, como já enfatizado anteriormente, é necessário avançar em outros aspectos do processo de rastreamento do câncer de mama (acesso a consultas; profissionais qualificados; tempo de espera entre consulta, diagnóstico e tratamento; monitoramento do estágio da doença; periodicidade do exame; campanhas de conscientização do público-alvo de rastreamento; entre outros condicionantes).

## Considerações finais

Os exames de rastreamento são considerados as principais estratégias para a detecção precoce de doenças quando não há sintomas aparentes. Em especial, a mamografia é uma importante ferramenta tendo em vista que o rastreamento eficaz aumenta as chances de detecção de um câncer de mama em estágio inicial que pode aumentar a sobrevivência e diminuir o sofrimento das pacientes.

Sob esse contexto, a atenção aos cuidados de rastreamento e detecção precoce se aliam às disparidades de acesso aos exames de mamografia pelo país. A literatura aponta a necessidade do aumento da cobertura das mamografias realizadas pelas mulheres ^3^ ^,^ ^11^ ^,^ ^12^ ^,^ ^15^. Uma das formas para o aumento dessa cobertura é a alocação de equipamentos entre diversas localidades ao redor do país. Considerando as especificidades de cada município, acredita-se que o efeito do recebimento de mamógrafos pode ser diferente entre as diversas cidades brasileiras. Dessa maneira, este estudo buscou analisar o efeito causal da alocação de mamógrafos pelo SUS a partir de 2014 sobre indicadores de saúde femininos.

Foram utilizados dados por residência disponíveis nos sistemas de informação do DATASUS para verificar se o número de municípios brasileiros que receberam o equipamento durante o período de 2014 a 2019 elevou o número de exames e diagnósticos realizados e reduziu a mortalidade por neoplasia maligna da mama. De forma geral, os municípios que obtiveram mamógrafos nesse período se concentram nas regiões Norte e Nordeste, com menor densidade demográfica e pouca concentração urbana. Recorreu-se à metodologia de pareamento por escore de propensão para o controle de variáveis observáveis e ao modelo de regressão por efeitos fixos de tempo e grupo para o controle de não observáveis fixas no tempo.

Os resultados indicaram que os municípios selecionados que tiveram mamógrafo em uso em algum momento a partir de 2014 obtiveram aumentos na realização de exames. As estimativas parecem ser bastantes claras em mostrar essa elevação, visto que os efeitos da presença dos mamógrafos foram positivos e altamente significativos nas estimações sem e com pareamento. Em contrapartida, as estimativas de curto prazo para os demais indicadores não foram significativas. A principal limitação deste estudo é o encontro de indicadores mais precisos a respeito dos diagnósticos e óbitos por neoplasia maligna da mama, já que a paciente pode ter seu óbito ou diagnóstico em local diferente de onde reside.

Ao levar em consideração o efeito causal sobre o aumento dos exames realizados, os achados deste estudo refletem a necessidade de otimizar o uso dos recursos e a disponibilidade de serviços e equipamentos de saúde para o rastreamento do câncer de mama em mais localidades. Além da utilização de mamógrafos, outros aspectos também afetam os indicadores de saúde e devem ser considerados na criação de políticas públicas de saúde, tais como profissionais especializados, acesso a consultas, subutilização dos equipamentos, distâncias geográficas entre o público-alvo e as unidades públicas de imagem ^12^, além de levar em consideração as características socioeconômicas das localidades prioritárias e fazer campanhas de conscientização da importância do rastreamento do câncer de mama na periodicidade bienal para mulheres de 50 a 69 anos ^6^ ^,^ ^27^.

Pesquisas futuras poderão analisar como esses mecanismos desencadeiam os efeitos causais da presença de equipamentos e a realização de exames sobre a redução da mortalidade e diagnósticos de câncer de mama. A continuidade de análises semelhantes são cruciais para o uso mais eficiente dos recursos disponíveis, a disponibilidade regional mais igualitária e a melhora da qualidade dos serviços de saúde oferecidos à população feminina.
